# Neurotensin Attenuates Nociception by Facilitating Inhibitory Synaptic Transmission in the Mouse Spinal Cord

**DOI:** 10.3389/fncir.2021.775215

**Published:** 2021-12-24

**Authors:** Ming-Ming Zhang, Yu-Peng Feng, Xin-Tong Qiu, Tao Chen, Yang Bai, Jia-Ming Feng, Jun-Da Wang, Yan Chen, Ming-Zhe Zhang, Hao-Kai Duan, Mingwei Zhao, Yi-Hui Teng, Jing Cao, Wei-Dong Zang, Kun Yang, Yun-Qing Li

**Affiliations:** ^1^Department of Anatomy, Histology and Embryology, K.K. Leung Brain Research Centre, The Fourth Military Medical University, Xi’an, China; ^2^Department of Anatomy, School of Medicine, Northwest University, Xi’an, China; ^3^Department of Anatomy, School of Medicine, Jiangsu University, Zhenjiang, China; ^4^Department of Anatomy, Basic Medical College, Zhengzhou University, Zhengzhou, China; ^5^Department of Anatomy, College of Basic Medicine, Dali University, Dali, China

**Keywords:** neurotensin, neurotensin receptor, nociception, GABA, mice, spinal dorsal horn

## Abstract

Neurotensin (NT) is an endogenous tridecapeptide in the central nervous system. NT-containing neurons and NT receptors are widely distributed in the spinal dorsal horn (SDH), indicating their possible modulatory roles in nociception processing. However, the exact distribution and function of NT, as well as NT receptors (NTRs) expression in the SDH, have not been well documented. Among the four NTR subtypes, NTR2 is predominantly involved in central analgesia according to previous reports. However, the expression and function of NTR2 in the SDH has not yet been directly elucidated. Specifically, it remains unclear how NT-NTR2 interactions contribute to NT-mediated analgesia. In the present study, by using immunofluorescent histochemical staining and immunohistochemical staining with *in situ* hybridization histochemical staining, we found that dense NT- immunoreactivity (NT-ir) and moderate NTR2-ir neuronal cell bodies and fibers were localized throughout the superficial laminae (laminae I-II) of the SDH at the light microscopic level. In addition, γ-aminobutyric acid (GABA) and NTR2 mRNA were colocalized in some neuronal cell bodies, predominantly in lamina II. Using confocal and electron microscopy, we also observed that NT-ir terminals made both close contacts and asymmetrical synapses with the local GABA-ir neurons. Second, electrophysiological recordings showed that NT facilitated inhibitory synaptic transmission but not glutamatergic excitatory synaptic transmission. Inactivation of NTR2 abolished the NT actions on both GABAergic and glycinergic synaptic release. Moreover, a behavioral study revealed that intrathecal injection of NT attenuated thermal pain, mechanical pain, and formalin induced acute inflammatory pain primarily by activating NTR2. Taken together, the present results provide direct evidence that NT-containing terminals and fibers, as well as NTR2-expressing neurons are widely distributed in the spinal dorsal horn, GABA-containing neurons express NTR2 mainly in lamina II, GABA coexists with NTR2 mainly in lamina II, and NT may directly increase the activity of local inhibitory neurons through NTR2 and induce analgesic effects.

## Introduction

Nociceptive information is transferred from the periphery to the spinal dorsal horn (SDH) or trigeminal nucleus caudalis dorsal horn neurons *via* primary afferent termination. Several classical amino acid neurotransmitters, such as glutamate, γ-aminobutyric acid (GABA) and glycine, as well as some neuropeptides, such as substance P (SP), calcitonin gene-related peptide (CGRP), and neurotensin (NT), are localized in the dorsal horn, indicating their possible roles in the transmission and modulation of nociception (Todd, 2010).

NT, consisting of thirteen amino acids (pGlu-Leu-Tyr-Glu-Asn-Lys-Pro-Arg-Arg-Pro-Tyr-Ile-Leu-OH), is an endogenous neuropeptide with a strong opioid-independent analgesic function (Dobner, 2006; Feng et al., 2015). In the SDH, NT-immunoreactive (NT-ir) cell bodies, fibers and terminals are found in the superficial laminae (laminae I and II) (Seybold and Elde, 1982; Difiglia et al., 1984; Liu et al., 2015). In accordance with the distribution pattern, NT plays important roles in modulating nociception as a neuromodulator (Martin and Naruse, 1982).

Four neurotensin receptor subtypes (NTR1-4) have been confirmed in the central nervous system (CNS). Among these subtypes, NTR1 and NTR2 are cloned and clarified as G-protein coupled 7-transmembrane receptors, while NTR3 and NTR4 are elusive and may exert functions unrelated to NT signaling (Vincent et al., 1999; Fassio et al., 2000; Sarret et al., 2003a,b). It has been previously indicated that NTR2 is predominantly involved in central analgesia (Kleczkowska and Lipkowski, 2013). However, its role has not yet been well elucidated at the spinal cord level. Our previous study showed that NTR2-immunoreactive rostral ventromedial medulla (RVM) neurons that project to SDH receive NTergic descending projecting fibers and terminals originating from the periaqueductal gray (PAG) (Wang et al., 2014). The morphological and functional analysis of NTR2 in the dorsal horn remains largely unknown.

Although it has been shown that NT facilitates glutamate release in dorsal horn neurons (Kadiri et al., 2011), it remains controversial whether NT facilitates or weakens inhibitory synaptic transmission. Therefore, in the present study, we first observed the distribution of NT, NTR2 and glutamic acid decarboxylase (GAD, a reliable biomarker for GABAergic neurons) in the SDH by using morphological methods. Then to investigate the morphological and physiological relationships between NTR2 and GABAergic signaling, we evaluated the regulatory effects of NT and NTRs antagonists on inhibitory synaptic transmission (especially GABAergic signaling) under nociceptive conditions by utilizing behavioral methods.

## Materials and Methods

### Animals

All procedures were approved by the Committee for Animal Care and Used for Education and Research of the Fourth Military Medical University (Xi’an, China).

The generation and characterization of the 67 kDa isoform of glutamic acid decarboxylase (GAD67)-GFP knock-in mice have been described previously (Tamamaki et al., 2003; Huang et al., 2013). Adult GAD67-GFP mice of both sexes were used for morphological study. Adult C57BL/6 wild-type mice of both sexes were used for electrophysiological studies and behavioral tests.

### Common Peroneal Nerve Ligation Model

The surgical methods were based on previous reports (Chen et al., 2014). Briefly, mice were anesthetized with 2% isoflurane. The common peroneal nerve (CPN) was visible between the anterior and posterior groups of thigh muscles, running almost transversely. The left CPN was slowly ligated with chromic gut sutures 5-0 until contraction of the dorsiflexor of the foot was visible as twitching of the digits. The skin was then sutured and cleaned. Sham surgery was conducted in the same manner, but the nerve was not ligated.

### Measurement of the Paw Withdrawal Mechanical Threshold

Measurements were based on previous reports (Chen et al., 2014). Mice were habituated to the testing environment for 3 days before baseline testing and then placed under inverted plastic boxes (7 × 7 × 10 cm) on an elevated mesh floor and allowed to habituate for 30 min before threshold testing. A logarithmic series of 8 calibrated Semmes-Weinstein monofilaments (von Frey hairs; Stoelting, Kiel, WI, United States) (0.008, 0.02, 0.04, 0.07, 0.16, 0.4, 0.6, 1, 1.4, and 2 g) with various bending forces (0.078, 0.196, 0.392, 0.784, 1.568, 3.92, 5.88, 9.8, 13.72, 19.6 mN) were applied to the plantar surface of the hind paw until the mice withdrew from the stimuli. Positive responses included licking, biting, and sudden withdrawing of the hind paws. A von Frey filament was applied 5 times to each testing area. The minimum bending force of the von Frey filament able to evoke 3 occurrences of the paw withdrawal reflex was considered the paw withdrawal threshold. All tests were performed in a double-blindmanner.

### Formalin Test

To test the analgesic effect of NT and the underlying mechanisms in SDH, a formalin pain model was employed. The animals were randomly divided into the NT group, the SR48692 + NT group and the SR142948A + NT group with 6 C57BL/6 mice in each group. After acclimating to the testing chamber surface for approximately 30 min, the mice were transiently anesthetized with isoflurane and subcutaneously (s.c.) injected into the plantar surface of the right hind paw with 10 μl of diluted 5% formaldehyde solution (dissolved in normal saline) or 10 μl of normal saline (sham control) using a syringe attached to a 30G needle. The mice with formaldehyde injection were carefully taken into the observing plastic chamber (10 × 10 × 10 cm), and a video was continually recorded for 60 min. The pain behaviors of the mice, including spontaneous flinches or licking of the injected hind paw, were manually recorded every 5 min, and the data were reviewed by analyzing the videos.

### Intrathecal Administration and Thermal Pain Behavioral Test

For intrathecal drug administration to the lumbar spinal cord, C57BL/6 mice were deeply anesthetized with 2% sodium pentobarbital (40 mg/kg, i.p.) and kept under proper anesthesia on a heating pad at 35°C with a temperature feedback device. The protocols were similar to those described elsewhere (Kleczkowska and Lipkowski, 2013), the middle line skin and muscle from L1 to S1 were cut, and a laminotomy was carried out to expose vertebrae between L5 and L6. With a 21G needle inserted, the dura matter was punctured, inferred by the backflow of clear spinal fluid. After removing the needle, a catheter (PE-10 tubing) was inserted into the subdural space and pushed rostrally. The catheter was tunneled under the skin and screwed over the head as an externalized connection. To avoid possible clogging, the catheter was flushed with 10 μl saline and sealed. The animals were routinely given analgesics and antibiotics for 3 days after surgery and housed for intrathecal administration. Only animals that totally recovered (without paresis or motor weakness) were used in further experiments. The NT (10 μg/μl in saline; from APE-BIO), NTR1 specific antagonist SR48692 (Buhler et al., 2005; Huang et al., 2008) (2-[[[1-(7-chloro-4-quinolinyl)-5-(2,6-dimethoxyphenyl)-1H-pyrazol-3-yl]carbonyl]amino]-tricyclo[3.3.1.13,7]decane-2-carboxylic acid) and NTR antagonist SR142948A (Binder et al., 2001) (2-[[5-(2,6-dimethoxyphenyl)-1-(4-(N-(3-dimethylaminopropyl)-N-methylcarbamoyl)-2-isopropylphenyl)-1H-pyrazole3-carbonyl]amino] adamantane-2-carboxylic acid hydrochloride) (both from APE-BIO) were intrathecally delivered with a 50-μl Hamilton syringe (both 10 μg/μl in 0.01% Tween 80 in saline). The unit conversion of drug concentration was adjusted according to previous studies.

Twenty-four intrathecally delivered mice and 12 control counterparts were used in the paw withdrawal latency (PWL) test to detect the baseline and drug effects. A heat radiant instrument (RTY-3; Xi’an Fenglan Instruments, China) was used to measure the sensitivity of mice hind paws to heat stimuli. Animals were laid on a glass surface under an inverted clear plastic chamber of a radiation thermal stimulator system and acclimatized for 30 min before the test. The plantar surface of each hind paw was impacted by a radiant heat stimulus from a projector lamp located under the glass floor. The lamp was cut off every 30 s to prevent tissue heat-burn.

### Electrophysiological Recordings

Acute spinal cord slice preparation was introduced in our previous work (Yang et al., 2001a). In brief, adult C57 mice (22–35 g) were deeply anesthetized by urethane (1.5–2.0 g/kg body weight, i.p.). After a laminectomy, the spinal cord trunk was carefully removed and immediately immersed in ice-cold (∼1°C) oxygenated artificial cerebrospinal fluid (aCSF) (in mM: NaCl 124, KCl 3.6, CaCl_2_ 2.5, NaH_2_PO_4_ 1.2, MgCl_2_ 1.2, NaHCO_3_ 25, and glucose 11; pH 7.2; 300–305 mOsm), saturated with a 95% O_2_/5% CO_2_. Under the dissecting microscope, the dura mater and the pia-arachnoid were carefully removed by fine forceps. The lumbosacral cord was mounted on a vibratome slicer (DTK-1000N; Dosaka, Kyoto, Japan) with an agar block support. Transverse slices (400 μm thickness) were excised from mouse spinal cord and incubated in aCSF (36°C) for 30 min. After incubation, slices were kept at room temperature (22–24°C) in aCSF for up to 8 h for electrophysiological recordings.

A single slice was transferred to a recording chamber (∼1 ml volume) with a perfusion rate of 2–3 ml/min. The recording electrodes were fabricated by a micropipette puller (P-97; Sutter Instruments, United States) with glass capillaries (TW150F-4; World Precision Instruments, United States). The lamina II layer of the SDH neurons can be found and clamped by using the conventional “blind patch” whole-cell voltage-clamp recording configuration. Either spontaneous excitatory postsynaptic currents (sEPSCs) at a holding potential of –70 mV or spontaneous inhibitory postsynaptic currents (sIPSCs) at a holding potential of 0 mV were recorded (Yang et al., 2001b). The sEPSCs and sIPSCs contained both action potential-dependent spontaneous events and action potential-independent miniature events. In the present study, miniature IPSCs (mIPSCs) were recorded in the presence of 0.5 μM tetrodotoxin (TTX) which blocked action potential-dependent presynaptic release. The intracellular internal solution for EPSCs contained (in mM): K-gluconate 135, KCl 5, CaCl_2_ 0.5, MgCl_2_ 2, EGTA 5, HEPES 5, and Mg-ATP 5 (pH 7.2 modulated by KOH, mOsm 290); the internal solution for IPSCs contained (in mM): Cs_2_SO_4_ 110, CaCl_2_ 0.5, MgCl_2_ 22, HEPES 20, TEA 5, and Mg-ATP 5 (pH 7.2 modulated by CsOH, mOsm 290). The liquid junction potential was not corrected. The resistance for recording pipettes was 6–8 MΩ when filled with internal solutions. According to the Nernst equation theory, the Cl^–^ for equilibrium potentials was –52 mM for the K-gluconate-based internal solution and –32 mV for the Cs_2_SO_4_-based internal solution. Using these internal solutions and with a –70 mV holding potential, the EPSCs were readily recorded without detectable IPSCs. On the other hand, at a 0 mV holding potential, IPSCs can be recorded without detectable EPSCs (Yang et al., 2001b).

According to the purposes of the experiments, GABAergic sIPSCs and glycinergic sIPSCs were recorded in the presence of strychnine (1 μM) and bicuculline (10 μM), respectively. The amplifier for data acquisition was Axopatch 200B, controlled by commercial software Clampex 10.2 (both from Molecular Devices, San Jose, CA, United States).

### Morphological Studies

#### Double Immunofluorescence Labeling

For double immunofluorescence labeling, six adult GAD67-GFP mice were perfused transcardially with 0.1 M phosphate buffer (PB; pH 7.4) containing 4% paraformaldehyde. Lumbar segments 3–5 of the spinal cord were obtained and postfixed with the same fixative for 4 h, placed in 30% (w/v) sucrose solution in 0.05 M PB solution (PBS; pH 7.4) overnight at 48°C and cut into 25-μm thick sections on a freezing microtome. The sections were incubated overnight at 4°C with a mixture of mouse anti-GFP antibody (1:1,000; Abcam) and rabbit anti-NT antibody (1:500; Abcam). The incubation medium was prepared by 0.01 M PBS (pH 7.4) containing 0.3% (v/v) Triton X-100, 0.12% (w/v) carrageenan, 1% (v/v) normal donkey serum, and 0.02% (w/v) sodium azide (PBS-XCD). After a rinse with PBS, the sections were incubated for 3 h at room temperature in PBS-XCD with a mixture of Alexa488-conjugated donkey anti-mouse antibody (1:500; Invitrogen) and Alexa594-conjugated donkey anti-rabbit antibody (1:500; Invitrogen). The sections were mounted onto gelatin-coated glass slides and cover-slipped with 50% (v/v) glycerol and 2.5% (w/v) triethylenediamine (antifading reagent) in 0.01 M PBS. The sections were observed under a confocal laser scanning microscope (FV-1000, Olympus, Japan) with a confocal depth of 1.0 mm.

#### *In situ* Hybridization Histochemistry

A DNA fragment complementary to mouse NTR2 cDNA was cloned into the pCRII vector (Invitrogen). Using these plasmids as templates, sense and antisense single-stranded RNA probes were synthesized with a digoxigenin (DIG) labeling kit (Roche Diagnostics, Germany). Six adult male GAD67-GFP mice were perfused transcardially with 0.1 M PB containing 4% paraformaldehyde. Lumbar segments 3–5 of the spinal cord were obtained and postfixed with the same fixative for 4 h, placed in diethylpyrocarbonate (DEPC)-treated 30% (w/v) sucrose solution in 0.05 M PBS overnight at 48°C and cut into 25 μm thick transverse sections on a freezing microtome. The sections were collected alternately in six sets of serial sections. After rinsing in PBS for 5 min twice, the sections were hybridized with 0.5 mg/ml DIG-labeled sense and anti-sense RNA probes for NTR2, respectively in a mixture of 50% (v/v) formamide, 75 mM NaCl, and 75 mM sodium citrate (5 SSC), 5 Denhardt’s solution, 250 mg/ml yeast tRNA, and 500 mg/ml salmon sperm DNA for 16–24 h at 60°C. After two washes in 2 SSC for 5 min each at 60°C, the sections were incubated for 30 min at 37°C with 10 mg/ml RNase A in a mixture of 0.5 M NaCl, 0.01 M Tris-HCl (pH 8.0), and 1 mM ethylene diamine tetra-acetic acid (EDTA) and then washed at RT in 2 SSC for 10 min. Subsequently, the sections were incubated with alkaline phosphatase (AP)-conjugated anti-DIG antibody Fab fragment (Roche Diagnostics), which was diluted at 1:2,000 with 0.1 M Tris-HCl (pH 7.5) containing 1% blocking reagent (Roche Diagnostics) and 0.15 M NaCl. Then, phosphatase was visualized by AP reaction for 20 h with 0.375 mg/ml nitroblue tetrazolium and 0.188 mg/ml 5-bromo-4-chloro-3-indolyl phosphate (NBT/BCIP, Roche Diagnostics), which were diluted at 1:50 with 0.1 M Tris-HCl containing 0.1 M NaCl and 5 mM MgCl_2_. The specificity of the hybridization reaction was verified by processing the same histological sections treated as described but with labeled sense probes instead of with antisense probes. Labeling was not observed under these conditions.

#### Double Labeling With *in situ* Hybridization Histochemistry and Immunohistochemistry

Following ISH, sections were further processed to reveal GFP immunoreactivity as described previously (Huang et al., 2008). The sections were incubated at RT overnight with rabbit anti-GFP antibody (1:1,000; Abcam) and further incubated in PBS-XCD at RT for 3 h with biotinylated anti-rabbit antibody (1:200; Merckmillipore). The sections were then incubated at RT for 3 h with avidin-biotinylated peroxidase complex (ABC-Elite kit, Vector, United States), which was diluted at 1:50 with 0.05 M PBS containing 0.3% (v/v) Triton X-100 (PBS-X). Finally, the bound peroxidase was developed by reaction with 0.02% (w/v) diaminobenzidine-4HCl (DAB) and 0.001% (v/v) H_2_O_2_ in 50 mM Tris-HCl (pH 7.6). The sections were mounted onto gelatin-coated glass slides, washed in water, dried, cleared in xylene, and cover-slipped.

#### Electron Microscopy

The procedures for immunoelectron microscopy have been described (Yang et al., 2001b). In brief, C57BL/6 mice were deeply anesthetized with an overdose injection of sodium pentobarbital (100 mg/kg, i.p.) and then perfused transcardially with 50 ml of 0.025 M PBS, followed by 200 ml of 0.1 M PB with a fixative containing 4% paraformaldehyde and 0.1% glutaraldehyde. Serial sections of the spinal lumbar enlargement were transversely cut at 40 μm thickness on a vibratome (DTK-1000N; Dosaka, Japan). Sections were divided into two series and collected. After cryoprotection, the sections were freeze-thawed with liquid nitrogen and then placed in 0.05 M Tris buffered saline (TBS) containing 20% normal sheep serum and incubated for 0.5 h to block non-specific immune responses.

After rinsing the sections with 0.05 M PBS, the first series of sections were subjected to GABA/NT immunocytochemistry at RT using the avidin-biotin complex (ABC) method. In brief, the sections were incubated with a mixture of mouse anti-GABA antibody (1:200; Sigma) and rabbit anti-NT antibody (1:200; Abcam) for 24 h followed by incubation with a mixture of donkey anti-rabbit IgG combined with biotin (1:200; Merckmillipore) and goat anti-mouse IgG combined with 1.4 nm immunogold particles (1:200; Nanoprobes) for 16–18 h. All sections were washed with 0.05 M TBS between the above two steps.

The following sections were fixed with 11% glutaraldehyde for 10 min, silver enhancement was performed for 8–14 min in an HQ Silver Kit (Nanoprobes, United States) and incubated in an ABC Kit (Vector) for 2 h. Sections were stained for 10–20 min in 0.05 M Tris-HCl buffer (pH 7.6) containing 0.02% diaminobenzidine and 0.003% hydrogen peroxide. Sections were washed with PBS 3 times for 10 min between two steps. After osmification, the sections were counterstained with uranyl acetate. With dehydration, the sections were mounted onto silicon-coated glass slides and then embedded in epoxy resin. Once the resin polymerized, section fragments that contained the superficial layer of the SDH were removed from the resin. The selected tissue fragments were further cut into 70 nm thick sections using an ultramicrotome (Reichert-Nissei Ultracut S, Leica), mounted on single-slot grids and examined with an electron microscope (JEM1440, Japan).

The second series of sections as a control group was not disposed of with the primary antibody, and a control experiment was performed employing the same staining steps as the first series. Under the electron microscope, the GABA-positive reaction product and NT immunogold labeling were not observed (data not shown).

### Data Analysis and Statistics

For morphological data analysis, the images were analyzed by using Leica Q500mc image processing (Leica Microsystems Inc., United States) or confocal laser scanning microscopy (FV-1,000, Olympus, Japan). For electrophysiological data analysis, for each neuron, the amplitudes and event distributions of sEPSCs and sIPSCs were detected by a created template with Clampfit 10.2 software (Molecular Devices) and analyzed with the Kolmogorov-Smirnov test (K-S test) to determine drug action (Yang et al., 2001a). The amplitudes and event distributions data for each cell were then grouped for paired *t*-test. For pain behavior tests, data were analyzed by comparing drug effects with the representative controls. Measured values for each group are expressed as the mean ± S.E.M. Student’s *t*-test was used for testing, and a *P*-value < 0.05 was considered to be significant. Paired *t*-tests were used to compare values before and during drug treatment; unpaired *t*-tests were used for group comparisons. SPSS software (version 21, IBM Inc., United States) was used for group comparisons.

## Results

### Intrathecal Neurotensin Attenuates the Heat Radiant Response Threshold

**FIGURE 1:**
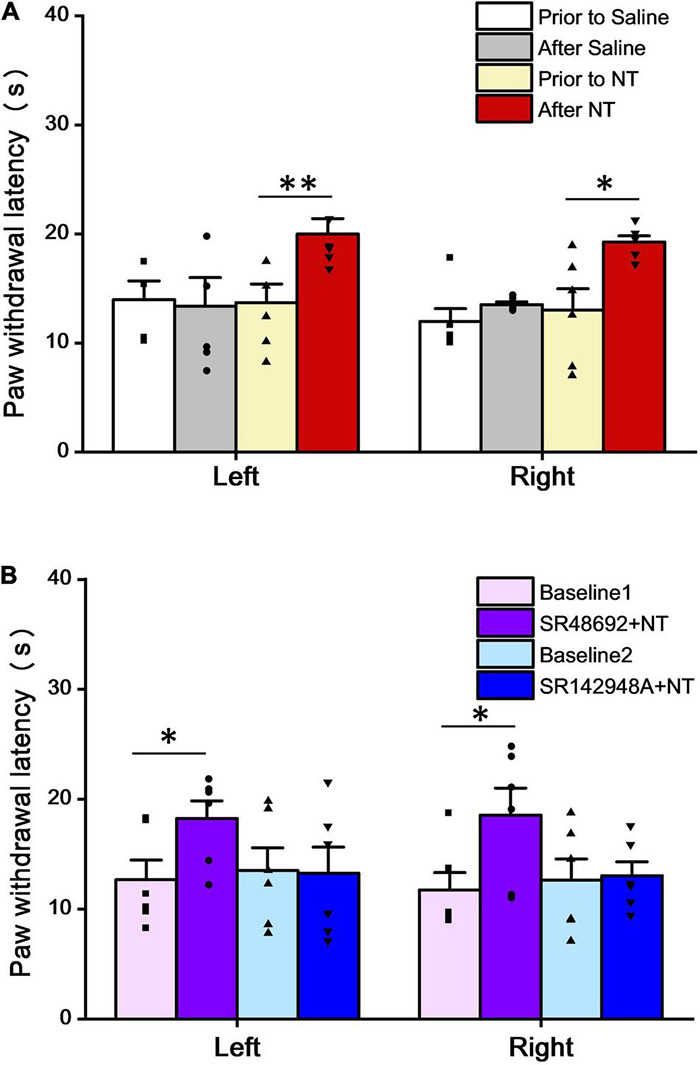
NT attenuated paw withdrawal latency (PWL) in the heat radiant test. **(A)** Intrathecal administration of NT (10 μg) significantly increased PWL in both the left and right hind paws, while saline had no effects. **(B)** Intrathecal coadministration of NT (10 μg) and SR48692 (10 μg), but not NT (10 μg), and SR142948A (10 μg), significantly increased PWL in both left and right hind paws, compared with baselines. Data are expressed as the mean ± S.E.M. *P < 0.05; ^**^P < 0.01; *n* = 6 per group, paired *t*-test.

The role of NT in the spinal cord level was studied by a heat radiant response test with 6 mice in each group. In the control group before saline injection, the PWL of the left hind paw was 13.97 ± 1.73 s, while that of the right hind paw was 11.97 ± 1.19 s (*n* = 6). Five minutes after saline injection, the left and right hind PWL to heat stimuli were 13.40 ± 2.22 and 13.53 ± 0.24 s, respectively (*P* > 0.05 in both left and right hind paws to the control, respectively; paired *t*-test), suggesting that saline injection has no significant effect on the heat pain responses. In the NT injection group (behavioral test was performed 5 min after intrathecal injection of 10 μl NT), the left hind paw had a threshold of 20.00 ± 1.40 s, while the right hind paw was 19.27 ± 0.58 s. Both groups were significantly longer than that before NT administration (*P* < 0.05 in both groups to the respective baselines, paired *t*-test) (Figure 1A). The results suggest that intrathecal application of NT could alleviate the thermal pain threshold in intact mice.

The next step was to determine which subtype of NTR was involved in this antinociceptive effect. In the SR48692 + NT group (behavioral test was performed 5 min after intrathecal injection of SR48692 and NT, dosing interval was 5 min), the PWL was 18.25 ± 1.62 and 18.57 ± 2.46 s in the left and right hind paws, respectively. Compared to the baselines (before SR48692 + NT treatment), 12.68 ± 1.79 s in the left hind paws and 11.75 ± 1.58 s in the right hind paws, both sides showed a significant PWL increase (*P* < 0.05 in both paired *t*-tests) (Figure 1B). Since SR48692 is a specific NTR1 antagonist, the results indicate that blockade of NTR1 does not affect NT antinociception in the thermal pain test.

However, in the SR142948A + NT group (behavioral test was performed 5 min after intrathecal injection of SR142948A and NT, dosing interval was 5 min), the baseline PWL for the left and right hind paws were 13.52 ± 2.07 and 12.63 ± 1.95 s, respectively. After SR142948A (an antagonist for both NTR1 and NTR2) + NT administration, the PWL was 13.27 ± 2.39 and 13.05 ± 1.27 s, respectively (Figure 1B). Combined with SR48692 + NT results, the data indicate that blockade of NTR2 invalidates NT analgesic effects in the thermal pain test. Taken together, the above data illustrate that NTR2 but not NTR1 is involved in NT antinociception in thermoalgesia at the spinal cord level.

### Intrathecal Neurotensin Attenuates Formalin Nociceptive Behaviors and Mechanical Allodynia

The formalin test was also used to further identify NT actions. All animals (6 mice in each group) exhibited typical biphasic nociceptive behaviors during the observation time (from formalin injection to 60 min). The whole formalin test responses were divided into phase 1 (0–10 min) and phase 2 (15–60 min), where phase 2 was further divided into phase 2A (15–30 min) and phase 2B (35–60 min). The effects of NT and NTR antagonists under different conditions were summarized in Tables 1, 2. Behavioral tests were performed 5 min after drugs intrathecal injection.

**TABLE 1:** Cumulated hind paw withdrawal times in the formalin test (phase 1 and phase 2).

Phases	i.t. Treatment	Withdrawal times	*P*-value compared to control
Phase 1 (0–10 min)	Saline (control)	77.83 ± 6.06	
	NT	53.83 ± 4.35	<0.05
	SR48692 + NT	69.00 ± 6.82	>0.05
	SR142948A + NT	60.33 ± 7.60	>0.05
Phase 2 (15–60 min)	Saline (control)	471.33 ± 14.61	
	NT	388.83 ± 25.17	<0.05
	SR48692 + NT	386.00 ± 22.08	<0.05
	SR142948A + NT	440.50 ± 29.57	>0.05
			

**TABLE 2:** Cumulated hind paw withdrawal times in the formalin (phase 2A and phase 2B).

Phases	i.t. Treatment	Withdrawal times	*P*-value compared to control
Phase 2A (15–30 min)	Saline (control)	202.00 ± 14 51	
	NT	120.17 ± 17.77	<0.05
	SR48692 + NT	121.33 ± 8.80	<0.05
	SR142948A + NT	196.83 ± 9.13	>0.05
Phase 2B (35–60 min)	Saline (control)	269.33 ± 17.72	
	NT	268.67 ± 24.19	>0.05
	SR48692 + NT	264.67 ± 18.74	>0.05
	SR142948A + NT	246.67 ± 27.94	>0.05

**FIGURE 2:**
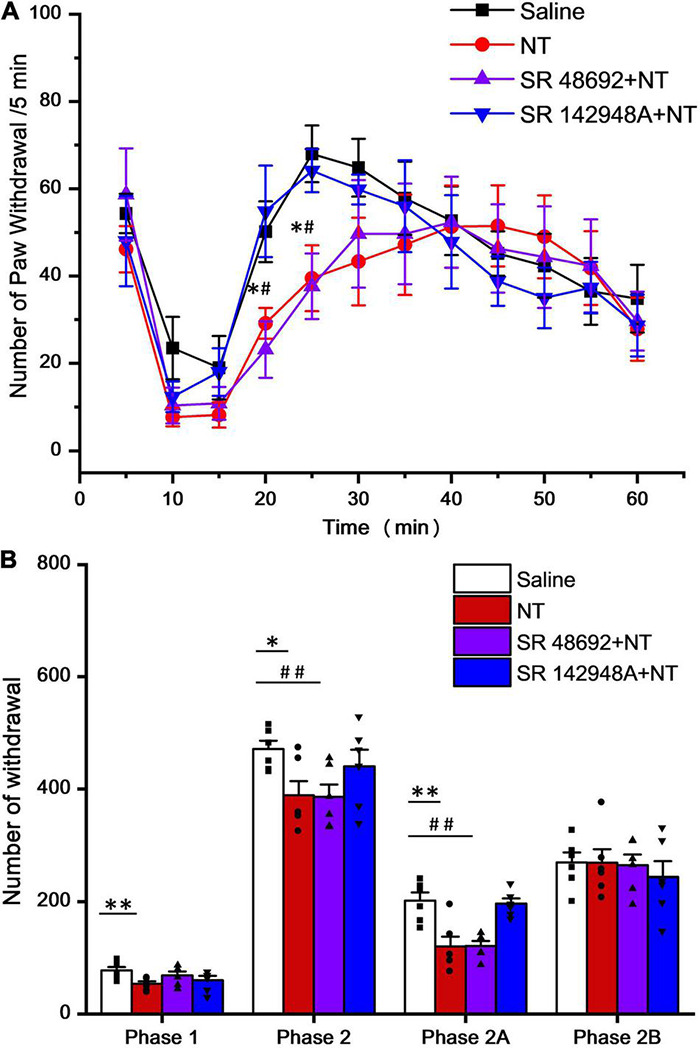
Time and agonist dependency of NT antinociceptive effects on intrathecal NT administration (hind paw retrieval times were counted every 5 min). **(A)** In phase 1 (0–10 min post formalin injection), all 4 groups showed no significant difference. In phase 2 (15–60 min post formalin injection), NT alone (red; *P < 0.05; compared to saline group, unpaired *t*-test) and SR48692 + NT (purple; ^#^*P* < 0.05; compared to saline group, unpaired *t*-test) significantly decreased paw retrieval times, while SR142948A + NT (blue) showed no change (*n* = 6 per group, unpaired *t*-test). **(B)** Column diagram further illustrates that NT exerts major analgesic effects in the early phase (phase 1 and phase 2A, 0–30 min post formalin injection), but not the late phase (phase 2B, 35–60 min post formalin injection), of the formalin induced pain (*P < 0.05; ^**^P < 0.01; compared to the saline group, unpaired *t*-test). SR142948A, but not SR48692, blocked NT’s analgesic effects. Data are expressed as mean ± S.E.M. ^#^*P* < 0.05; ^##^*P* < 0.01; compared to the saline group, *n* = 6 per group, unpaired *t*-test.

In formalin pain model mice, the acute nociceptive response hind paw withdrawal times were significantly decreased by NT pretreatment, indicating that NT administration largely attenuated nociceptive responses in phase 1. Both NTR1 and NTR2 were involved in nociception in this early phase (Table 1 and Figure 2). While this action was blocked in either the SR498692 + NT group or the SR142948A + NT group, indicating that NTR1 was involved in NT-mediated antinociception in this phase, the possibility of NTR2 playing a role in phase 1 cannot be excluded. NTRs are not involved in acute pain in the present model. In phase 2 (inflammatory pain response), NT alone significantly attenuated paw retrieval times. However, SR498692 + NT administration but not SR142948A + NT administration attenuated NT action, further indicating that NTR2 was involved in antinociceptive effects in phase 2.

In detail, 5 min after intrathecal injection of NT (10 μg/μl), the times for observing spontaneous flinches or licking of the injected hind paw were accounted for every 5 min (Figure 2A). In phase 2A, NT treatment significantly attenuated pain behaviors and this effect did not involve NTR1 because coadministration of SR48692 and NT did not block the effect. However, blocking NTR2 with SR142948A abolished the NT action, indicating that NTR2 was involved in phase 2A. In phase 2B, NT did not show an antinociceptive effect, and blocking either NTR1 by SR48692 or NTR1 and NTR2 by SR142948A did not produce an analgesic effect (Table 2 and Figure 2B), indicating that NT exerted no analgesic effect in the late phase of the formalin test.

**FIGURE 3:**
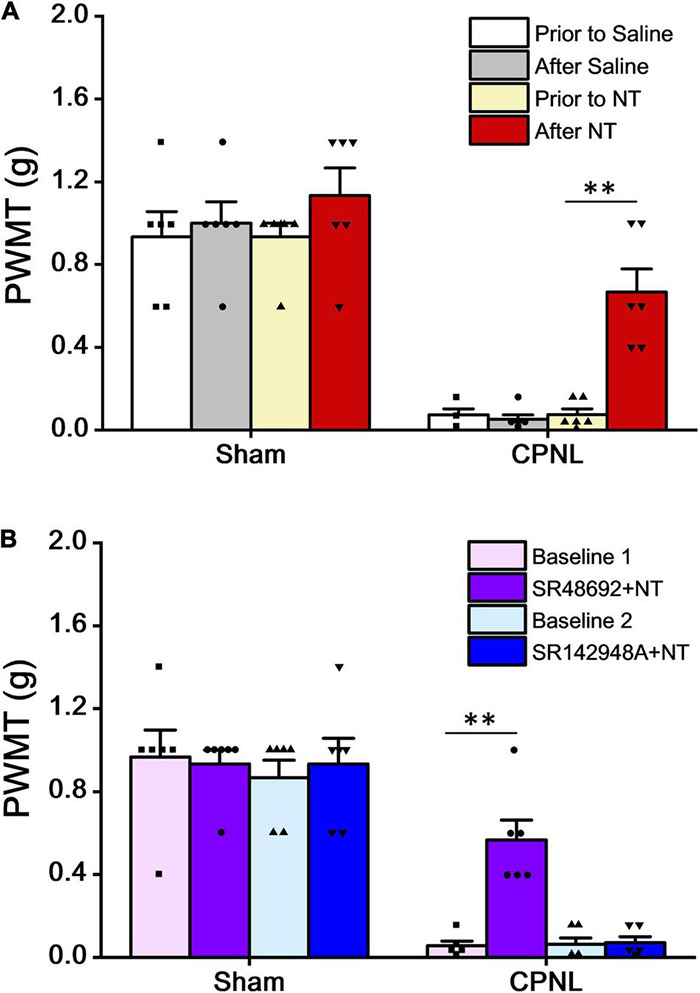
NT alleviated the paw withdrawal mechanical threshold (PWMT) in mice with CPNL. **(A)** Intrathecal administration of NT (10 μg) significantly increased PWMT in CPNL mice (^**^P < 0.01, compared with prior to NT group, *n* = 6 per group, paired *t*-test). **(B)** Intrathecal coadministration of SR48692 (10 μg) and NT (10 μg), but not SR142948A (10 μg) and NT (10 μg), significantly increased PWMT in CPNL mice, compared with baseline. ^**^P < 0.01; *n* = 6 per group, paired *t*-test.

In the CPNL group, the left hind paw withdrawal mechanical threshold (PWMT) was tested on day 7 after sham surgery or nerve ligation. The results showed that PWMT in CPNL mice was significantly decreased by NT treatment (PWMT were 0.07 ± 0.03 and 0.67 ± 0.11 g before and 5 min after NT administration, respectively. *P* < 0.001, paired *t*-tests), indicating that NT administration attenuated nociceptive responses effectively (Figure 3A). We also tested antagonists’ effects on PWMT. Our data showed that blockade of NTR1 had no impact on NT antinociceptive effects in mice with CPNL (PWMT were 0.06 ± 0.02 and 0.57 ± 0.10 g in the baseline 1 and SR48692 + NT groups, respectively. *P* < 0.001, paired *t*-tests) (Figure 3B). These results were consistent with thermal pain and formalin induced inflammatory pain tests.

### Neurotensin Facilitates Inhibitory but Not Excitatory Synaptic Transmission

**FIGURE 4:**
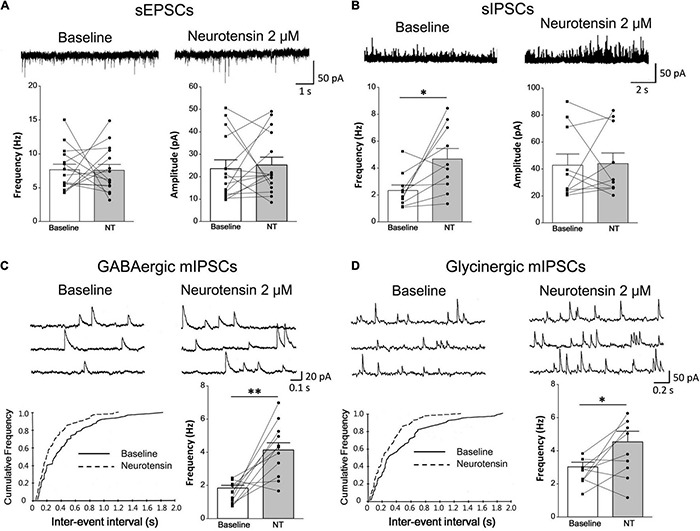
Pharmacological effects of NT on synaptic transmission in lamina II neurons of the SDH. **(A)** Comparison between baseline and NT treatment groups showed no significant changes in sEPSCs (*n* = 15 neurons). **(B)** GABAergic sIPSCs were recorded continuously at baseline and in the presence of 2 μM NT (*n* = 10 neurons). Comparison between baseline and NT treatment groups showed that NT increased the frequency of sIPSCs. **(C,D)** NT application to the bath increased frequency of GABAergic mIPSCs (*n* = 11 neurons) and glycinergic mIPSCs (*n* = 9 neurons). The upper traces show original recordings and the lower charts show K-S test which indicates significant left shifts in both GABAergic **(C)** and glycinergic **(D)** event distributions. The histograms show comparison between baseline and NT treatment groups. *P < 0.05; ^**^P < 0.01, paired *t*-test.

Inhibitory synaptic transmission in the dorsal horn plays important roles in modulating nociception from the peripheral nervous system to the central nervous system (Boyle et al., 2017). The whole-cell voltage-clamp recordings on spinal lamina II neurons were performed to study the effect of NT on neurotransmitter release by monitoring either sEPSCs or sIPSCs. NT perfusion (2 μM, 3 min) in the bath to the recording chamber did not influence the sEPSC amplitude or frequency on most neurons (baseline: 23.59 ± 3.62 pA and 7.66 ± 0.81 Hz; in the presence of NT: 25.25 ± 3.45 pA and 7.57 ± 0.87 Hz; *P* = 0.75 for amplitude and *P* = 0.94 for frequency, paired *t*-test) (Figure 4A). At a holding potential of 0 mV, NT increased the frequency of GABAergic sIPSCs (baseline: 2.34 ± 0.41 Hz; in the presence of NT: 4.68 ± 0.78 Hz; *P* < 0.05, paired *t*-test), with no significant effect on its amplitude (baseline: 23.59 ± 3.62 pA; in the presence of NT: 25.25 ± 3.45 pA; *P* > 0.05, paired *t*-test) (Figure 4B). Since spontaneous IPSCs contained both presynaptic action potential-dependent and action potential-independent events, we further analyzed miniature events (i.e., mIPSCs) in the presence of 0.5 μM tetrodotoxin (TTX). As shown by representative neurons and pooled data in Figures 4C,D, the frequency of both GABAergic and glycinergic mIPSCs significantly increased with NT treatment (baseline for GABAergic and glycinergic mIPSCs: 1.84 ± 0.16 and 3.03 ± 0.28 Hz; in the presence of NT for GABAergic and glycinergic mIPSCs: 4.14 ± 0.43 and 4.54 ± 0.64 Hz; *P* < 0.05 for both groups, paired *t*-test). The amplitude of both GABAergic and glycinergic mIPSCS did not change under the condition of NT treatment (baseline for GABAergic and glycinergic mIPSCs: 36.50 ± 3.37 pA and 69.16 ± 6.09 pA; in the presence of NT for GABAergic and glycinergic mIPSCs: 36.71 ± 3.63 pA and 70.47 ± 6.38 pA; *P* = 0.97 for GABAergic and *P* = 0.89 for glycinergic, paired *t*-test). The results suggest that NT specifically increases inhibitory but not excitatory synaptic transmission in the spinal dorsal horn.

**FIGURE 5:**
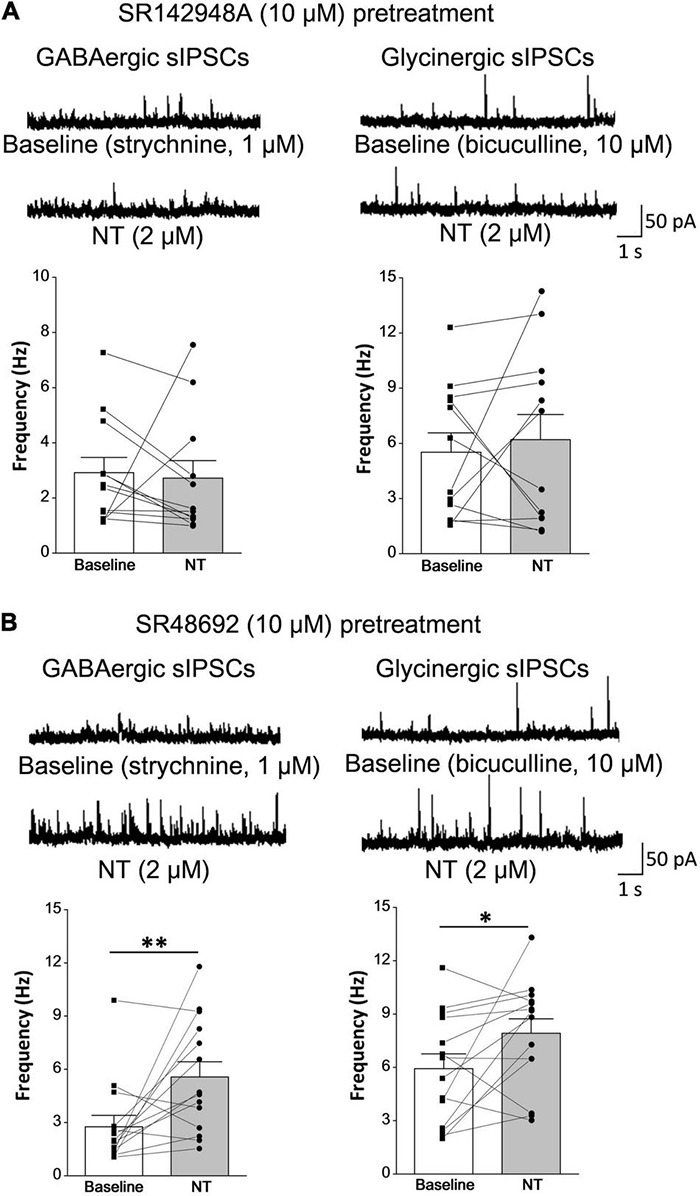
NT facilitates inhibitory synaptic transmission in lamina II neurons via the NTR2 subtype. **(A)** NTR antagonist SR142948A pretreatment (10 μM, 5 min) abolished NT action on GABAergic and glycinergic sIPSCs (*n* = 12 neurons). **(B)** Pretreatment with the NTR1 antagonist SR48692 (10 μM, 5 min) did not prevent GABAergic or glycinergic sIPSC frequency increases induced by NT perfusion (*n* = 14 neurons). *P < 0.05; ^**^P < 0.01, paired *t*-test.

To distinguish the role of NTR1 and NTR2 subtypes, SR48692 (a specific antagonist of NTR1) and SR142948A (an antagonist for both NTR1 and NTR2) were tested on NT action. SR142948A pretreatment (10 μM, 5 min) abolished NT action on GABAergic sIPSCs (baseline: 2.92 ± 0.56 Hz, in the presence of NT: 2.72 ± 0.63 Hz; *P* = 0.80, paired *t*-test). But SR48692 pretreatment (10 μM, 5 min) had no significant effect on NT action (baseline: 2.76 ± 0.64 Hz, in the presence of NT: 5.57 ± 0.86 Hz; *P* < 0.01, paired *t*-test), suggesting that NT enhanced inhibitory neurotransmitter GABA release by activating NTR2 (Figures 5A,B, left). In addition, NT slightly increased the frequency of glycinergic sIPSCs, which could be abolished by SR142948A (baseline: 5.52 ± 1.04 Hz, in the presence of NT: 6.20 ± 1.37 Hz; *P* > 0.05, paired *t*-test) but not SR48692 (baseline: 5.93 ± 0.83 Hz, in the presence of NT: 7.92 ± 0.81 Hz; *P* < 0.05, paired *t*-test) (Figures 5A,B, right). In sEPSC and sIPSC recordings, CNQX (10 μM), bicuculline (10 μM), and strychnine (1 μM) were applied to block glutamatergic sEPSCs, GABAergic sIPSCs, and glycinergic sIPSCs, respectively, confirming their respective natures (Yang et al., 2001a). Taken together, the data suggest that NT might exert an analgesic effect at the spinal cord level. The mechanism of this effect might be that NT enhances the release of inhibitory neurotransmitters, resulting in the inhibition of nociceptive information transmission in the spinal dorsal horn.

### Distributions of Neurotensin- and NTR2-Immunopositive Structures in the Spinal Dorsal Horn

**FIGURE 6:**
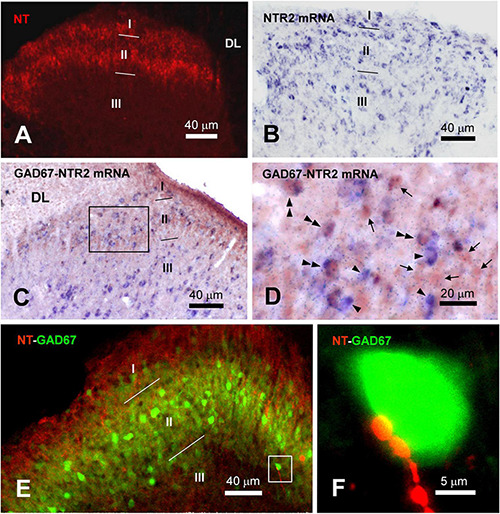
Distributions of NT-ir structures and NTR2-ir neurons and their colocalizations and connections in the spinal dorsal horn. **(A)** NT-ir neuronal cell bodies, fibers and terminals (red) in the superficial laminae (I–II) of the spinal dorsal horn (SDH) revealed by immunofluorescent histochemistry. **(B)** NTR2 mRNA positive neuronal cell bodies (blue) in laminae I–III of the SDH exhibited by *in situ* hybridization histochemistry. **(C)** The results of immunohistochemistry combined with *in situ* hybridization histochemical double-labeling showed that GABA (brown) coexisted with NTR2 mRNA (blue) in many neurons observed in lamina II of the SDH. **(D)** High magnification of the rectangular area in **(C)** shows the distributions of GABA-ir neurons (arrows), NTR2 mRNA-positive neurons (single arrowheads) and GABA/NTR2 mRNA coexisting neurons (double arrowheads) mainly observed in lamina II. **(E)** Confocal scanning images showing the distributions of NT-ir neuronal cell bodies, fibers and terminals (red) and GABA-ir neurons (green) revealed by immunofluorescence histochemical double-staining. **(F)** High magnification of the rectangular area in **(E)** indicated the close connection between the NT-ir terminals and GABA-ir neuronal cell bodies. DL, dorsal funiculus.

In the spinal dorsal horn, dense NT-ir fibers and terminals were found throughout the superficial laminae I and II but fainter in the deep laminae (Figure 6A). ISH data indicated a vast distribution of NTR2 mRNA immunostained cell bodies in the dorsal horn (Figures 6B–D). In specific, NTR2-ir cell bodies and terminals were scattered in the superficial dorsal horn with a higher expression in the lateral part of the whole dorsal horn. The highest NTR2 was found in lamina II with scattered somas (number of NTR2-ir neurons were 16 ± 3 in lamina I, 78 ± 7 in lamina II, 25 ± 4 in lamina III; *n* = 9 sections from 3 mice) and dense neuropils (Figure 6B).

### NTR2-ir mRNA Co-expression Within γ-Aminobutyric Acid-Containing Neurons

To confirm the correlation of GABAergic neurons with NT-ir fibers, combined immunofluorescent histochemical staining for GAD67 and ISH for NTR2 mRNA were performed. In the superficial dorsal horn, primarily in lamina II, NTR2 mRNA and GAD67 coexpressed cell bodies. As shown in Figures 6C,D, GAD67-ir somas (red brown) were scattered in the superficial dorsal horn, while NTR2 mRNA (blue) with variable soma sizes was scattered in the dorsal horn. Under the light microscope, double-staining of NTR2 mRNA and GAD67 demonstrated both brown and blue soma immunoreactivity (Figures 6C,D). Sixty-seven of 292 (22.95%) GAD67-ir somas in lamina II showed NTR2 mRNA positivity, while 38 of 87 (43.68%) NTR2 mRNA was GAD67-ir in stained sections. In accordence with the ISH results, immunofluorescence histochemical staining also showed colocolization of NTR2 and GAD67 in the SDH (Supplementary Figure 1). Since GAD67 is the major GABA synthesizing enzyme and is used as a proxy for GABAergic neurons, the data suggest that NTR2 exerts physiological effects mainly through inhibitory interneurons in the spinal cord.

### NT-Immunoreactivity Terminals Contact GABAergic Neurons in the Superficial Dorsal Horn

As reported previously (Todd, 2010), GABA-ir soma and terminals were mainly confronted in lamina II. Confocal microscopy scanning showed that, NT-ir fibers and terminals made close contact with GABAergic neurons (indicated by GAD67) in lamina II, which was shown by overlapping yellow spots (Figure 6E). In some cases, one GABAergic soma had multiple overlapping spots with NT-ir terminals, suggesting that NT might exert regulating effects on GABAergic neurons (Figure 6F).

**FIGURE 7:**
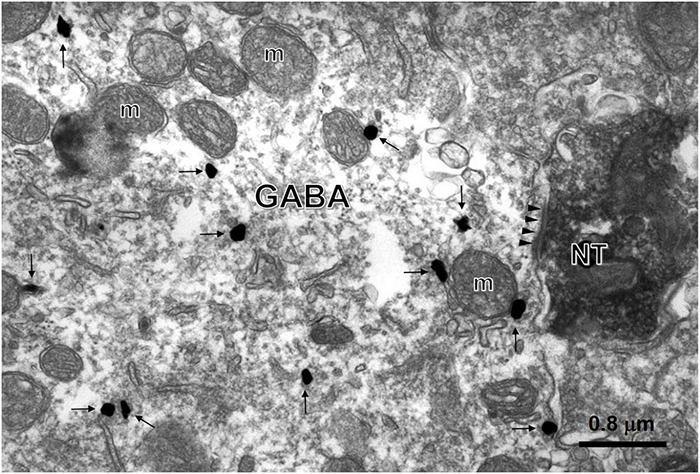
Synaptic connections between NT-ir terminals and GABA-ir neurons. An asymmetric synapse (triangles) between the NT-ir terminal (NT) tagged by black DAB reaction products and the GABA-ir large dendrite labeled by black gold nanoparticles (arrows) enhanced by a silver enhancement kit was found in lamina II of the SDH (m, mitochondrion).

To investigate whether NT terminals and GABAergic neurons were synaptically connected, we next carried out an EM study. NT-positive products were characterized by the presence of electron-dense DAB reaction products within axon terminals in laminae II, most of which were filled with small, round, black and clear synaptic vesicles. GABA-ir profiles were ultrastructurally determined by the presence of immunogold-silver grains (black round or oval particles with high-electron densities) distributed in the cytoplasm of the dendrites (Figure 7). Within lamina II, NT-ir terminals made asymmetrical axon-dendritic synapses with GABA-ir dendritic processes. Forty-two of the 285 GABA-ir neurons from 20 sections had been in contact with NT-ir terminals on their dendrites and neuronal cell bodies. The results suggest that NT may play an inhibitory role in the processing of pain by modulating GABA release in the SDH.

## Discussion

**FIGURE 8:**
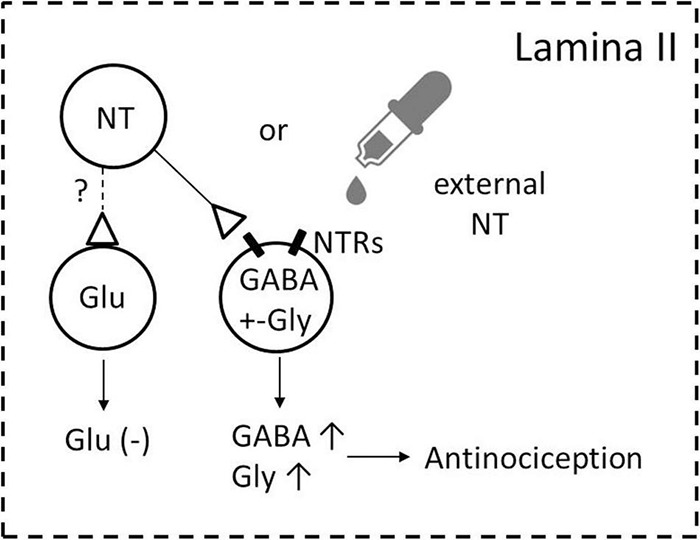
Schematic illustration of the possible ways through which NT exerts analgesia in the SDH. NT-containing neurons and NTR/GABA coexisting neurons are densely found in lamina II of the SDH. Activating NTRs by either endogenous NT (from NT-containing neurons in the SDH) or exogenous NT (NT administration to the dorsal horn and/or released from descending NTergic projections originating from the brainstem) induces increasing GABA release (GABA↑), and the increasing release of GABA subsequently modulates nociception (antinociception) either on primary afferent fibers (Zhang et al., 1995) and/or dorsal horn projection neurons to the supraspinal structures (Seybold and Maley, 1984). Whether NT activates glutamate-containing excitatory neurons remains to be determined (shown by the broken line and question mark). A possible NT action on descending fibers is not included in the present cartoon.

The major results of the present study are as follows: (1) there are dense NT-ir neuronal cell bodies and fibers and moderate NTR2-ir in the superficial layers of the SDH; (2) NT exerts analgesia in thermal pain and tissue-injury pain models, at least partially due to excitation of the inhibitory neurons; and (3) NT-ir terminals make synapses with GABA-containing neurons. Taken together, the results lead to the conclusion that NT-ir and NTR2 are widely distributed in the SDH, and the released NT might directly modulate the activity of inhibitory neurons (especially GABAergic neurons) and result in antinociceptive action (Figure 8). The mechanism of this antinociceptive effect might be that NT activates NTR2-expressing GABAergic neurons and in turn, enhances the presynaptic release of the inhibitory neurotransmitters.

### NT-Immunoreactivity and NTR2-ir in Spinal Dorsal Horn

NT-containing cell bodies and fibers are heterogeneously distributed throughout the pain modulation system, including the dorsal horn (Kalivas et al., 1982; Seybold and Elde, 1982; Shipley et al., 1987; Wang et al., 2014; Liu et al., 2015). The SDH neurons receive nociceptive messages from the periphery and transmit them to higher levels during these processing events, and the nociception in the dorsal horn receives multiple modulations from descending axons and local interneurons, making the dorsal horn a pivotal site for nociception processing and modulation (Todd, 2010). In addition to “classic” neurotransmitters, peptides (including NT) are also involved in nociception transmission and modulation (Fürst, 1999; Gonzalez-Suarez and Nitabach, 2018). Despite extensive studies on NT distribution in the spinal cord, previous reports have provided little information about the organization of NT-ir structures and their relationships with GABAergic neurons. The present study found that a “two-band” expression pattern is exhibited by NT-containing neurons in the dorsal horn, with overlap to IIo and IIi. These results are in line with the findings of previous studies (Jennes et al., 1982; Kalivas et al., 1982; Faull et al., 1989). Since lamina II is mainly composed of interneurons with neurochemically distinct populations, the heterogeneous distribution patterns suggest that NT may have specific roles in certain subtype neurons (Todd, 2010, 2017; Boyle et al., 2017; Gutierrez-Mecinas et al., 2017).

### Neurotensin Modulation on GABAergic Neurons

Although it has been well demonstrated that NT exerts analgesia *via* the descending serotonin system at the spinal cord level, the underlying mechanisms of NT at SDH remain elusive (Mileti and Randi, 1979; Kadiri et al., 2011; Wang et al., 2014). Direct administration of NT or its analogs predominantly excites dorsal horn neurons to either no-noxious or noxious stimuli from the periphery; thus, it is difficult to draw a conclusion that NT directly affects nociception from the peripheral to the spinal cord (Stanzione and Zieglgnsberger, 1983; Fürst, 1999). Since dorsal horn neurons release multiple inhibitory neurotransmitters including GABA and glycine, it is conceivable that NT activates inhibitory transmission (Todd, 2010). In fact, the results of this study provide convincing evidence that NT facilitates inhibitory but not excitatory synapse transmission (see Figure 4). A previous report showed that NT induced both pre- and postsynaptic activation in SG neurons (Kadiri et al., 2011), which was somewhat different from our electrophysiological and morphological study. This discrepancy might be attributed to neurodevelopment, since the previous study was based on physiological recordings of cultured neonatal (3–4-day-old) rat dorsal horn neurons. In addition, the dose of NT used in cultured neurons was 100 nM, which was significantly lower than that used in the present study. To clarify this issue, further experiments are required.

The present study provides evidence that NT increases the inhibitory neurotransmitter release in the SDH. We also observed that NTR2 mRNA is expressed on GABA-containing neurons. Taken together, these data support the notion that NT influences GABA release via presynaptic NTR2. It is worth noting that GABA and NT do not coexist in the SDH neurons (Todd et al., 1992; Proudlock et al., 1993); however, this property does not exclude the possibility that NT-ir terminals contact GABA-containing neurons. Since the NT facilitation effect has also been seen on glycinergic synaptic transmission, more morphological and functional studies are needed in the future.

### Neurotensin Receptor Subtypes Involved in Neurotensin Analgesia

Among the four subtypes of NTRs, the antinociceptive functions of NTR3 and NTR4 are still obscure (Pettibone et al., 2002; Remaury et al., 2002). It has been suggested that NTR1 is involved in NT analgesia (Breton et al., 2008), and NTR2 also plays important roles in previous reports (Sarret et al., 2003b; Lafrance et al., 2010). In our present study, using receptor subtype-preferring antagonists, we found that NTR1 and NTR2 played different roles in antinociception: NT exerted antinociceptive effects in heat pain *via* NTR2 but not NRT1. On the other hand, in the formalin test, phase 1 antinociception was mediated by both NTR1 and NTR2, while analgesia in phase 2 was mediated by NTR2. Our electrophysiological data further supported a critical role of NTR2 in mediating NT antinociception in heat pain and formalin pain, while NTR1 was involved in antinociception in phase 1 of the formalin nociceptive response, making NTR2 a promising target and its analogs promising analgesics. Breton et al. (2008) reported that in the spinal cord, NTR1-preferring agonists induced a dose-dependent reduction in Fos-like immunoreactive neurons, which was in line with our results, indicating that NTR1 is involved in the early phase of the formalin test. Taken together, both NTR1 and NTR2 were involved in NT-induced analgesia in our present study.

### Functional Significance

Since the discovery of its analgesic effects, NT has attracted particular interest because it is as potent as opioids at the same dose. NT-induced analgesia has been confirmed by multiple physiological and behavioral studies (Dubuc et al., 1994; Smith et al., 1997; Gui et al., 2004; Buhler et al., 2005; Breton et al., 2008). In the present study, by combining morphological, electrophysiological and behavioral methods, dense NT-ir terminals in the dorsal horn, the modulation effect of NT on inhibitory synapses, and the antinociceptive effect of NT in heat pain and tissue injury pain were identified. More information and mechanisms were added to indicate that NT and NTR2 are prospective candidates for new analgesics. A simplified mechanism underlying the analgesic action of NT is suggested in Figure 8.

It was reported that NT exerted antinociceptive effects at the spinal cord level, partially via presynaptic inhibition on the primary afferent central terminals (Zhang et al., 1995, 1996). In the present study, our morphological evidence shows that NT-ir terminals made synaptic connections with GABAergic neurons and therefore modulated inhibitory synapses. We cannot exclude the possibility that NT acts directly on the primary afferent central terminals and thus modulates nociception processing at the dorsal horn.

In summary, NT is released in the dorsal horn, and this peptide may act as a neurotransmitter and has promising antinociceptive functions through presynaptic activation of inhibitory neurons. Possessing specific opioid-independent antinociceptive effects in the SDH local circuit, NT and its analogs warrant further investigation according to the present study.

## Data Availability Statement

The original contributions presented in the study are included in the article/Supplementary Material, further inquiries can be directed to the corresponding author/s.

## Ethics Statement

The animal study was reviewed and approved by the Committee for Animal Care and Used for Education and Research of the Fourth Military Medical University (Xi’an 710032; China).

## Author Contributions

Y-QL, KY, and M-MZ conceived the project and designed the experiments. M-MZ, Y-PF, and X-TQ performed the animal experiments and behavior tests. J-MF, J-DW, YC, Y-HT, and JC completed morphological staining. TC, YB, MZ, M-ZZ, and H-KD performed the electrophysiological study. M-MZ, Y-PF, KY, and Y-QL drafted the manuscript. W-DZ, KY, and Y-QL designed and finished the final version of the manuscript. All authors read and approved the final manuscript.

## Conflict of Interest

The authors declare that the research was conducted in the absence of any commercial or financial relationships that could be construed as a potential conflict of interest.

## Publisher’s Note

All claims expressed in this article are solely those of the authors and do not necessarily represent those of their affiliated organizations, or those of the publisher, the editors and the reviewers. Any product that may be evaluated in this article, or claim that may be made by its manufacturer, is not guaranteed or endorsed by the publisher.

## Supplementary Material

Supplementary Figure 1 Distributions of NTR2 and GAD67 in the spinal dorsal horn. Confocal scanning images showed the distributions of GAD67-ir neuronal cell bodies, fibers, and terminals (green, **A,D**) and the NTR2-ir neuronal cell bodies (red, **B,D**), as well as DAPI-ir cell nucleus (blue, **C,D**) in the spinal dorsal horn (SDH) as revealed by immunofluorescence histochemical triple-staining. High magnification **(E)** of the rectangular area in **(D)** indicated the colocalization and connections of NTR2-ir and GAD67-ir structures in the SDH.
