# Supplementary material for: Neurotensin Attenuates Nociception by Facilitating Inhibitory Synaptic Transmission in the Mouse Spinal Cord
Source: Front Neural Circuits. 2021 Dec 24;15:775215. doi: 10.3389/fncir.2021.775215 (PMC8740200; doi:10.3389/fncir.2021.775215)
Supplement: Supplementary Figure 1 — Distributions of NTR2 and GAD67 in the spinal dorsal horn. Confocal scanning images showed the distributions of GAD67-ir neuronal cell bodies, fibers, and terminals (green, A,D) and the NTR2-ir neuronal cell bodies (red, B,D), as well as DAPI-ir cell nucleus (blue, C,D) in the spinal dorsal horn (SDH) as revealed by immunofluorescence histochemical triple-staining. High magnification (E) of the rectangular area in (D) indicated the colocalization and connections of NTR2-ir and GAD67-ir structures in the SDH. [file Table_1.docx]

**Row data of Fig.1-5**

| **Fig. 1A** | mice | Left PWL (s) | Right PWL (s) |
| --- | --- | --- | --- |
| Prior to Saline | 1 | 19.9 | 10.8 |
|  | 2 | 15.4 | 17.8 |
|  | 3 | 17.5 | 10.8 |
|  | 4 | 10.2 | 11.7 |
|  | 5 | 10.5 | 10.6 |
|  | 6 | 10.3 | 10.1 |
| After Saline | 1 | 19.3 | 14.4 |
|  | 2 | 15.2 | 14.1 |
|  | 3 | 19.8 | 13 |
|  | 4 | 9.1 | 13.1 |
|  | 5 | 9.6 | 13.4 |
|  | 6 | 7.4 | 13.2 |
| Prior to NT | 1 | 18.8 | 18.9 |
|  | 2 | 17.5 | 16.9 |
|  | 3 | 10.1 | 12.6 |
|  | 4 | 12.4 | 7.9 |
|  | 5 | 15.2 | 14.8 |
|  | 6 | 8.2 | 7.1 |
| After NT | 1 | 26.3 | 21.2 |
|  | 2 | 21.4 | 18.1 |
|  | 3 | 16.8 | 20 |
|  | 4 | 18.7 | 19.6 |
|  | 5 | 18.9 | 19.5 |
|  | 6 | 17.9 | 17.2 |

| **Fig. 1B** | mice | Left PWL (s) | Right PWL (s) |
| --- | --- | --- | --- |
| Baseline1 | 1 | 11.4 | 9.8 |
|  | 2 | 18.3 | 18.8 |
|  | 3 | 18.1 | 9.7 |
|  | 4 | 10.2 | 13.8 |
|  | 5 | 8.3 | 9.3 |
|  | 6 | 9.8 | 9.1 |
| SR48692+NT | 1 | 14.4 | 11.4 |
|  | 2 | 20.9 | 21.1 |
|  | 3 | 20.6 | 19.1 |
|  | 4 | 21.8 | 23.9 |
|  | 5 | 19.6 | 24.8 |
|  | 6 | 12.2 | 11.1 |
| Baseline2 | 1 | 12.3 | 14.6 |
|  | 2 | 7.8 | 16.9 |
|  | 3 | 19.8 | 9.2 |
|  | 4 | 8.6 | 18.8 |
|  | 5 | 19.1 | 9.1 |
|  | 6 | 13.5 | 7.2 |
| SR142948A+NT | 1 | 17.5 | 9.5 |
|  | 2 | 9.6 | 10.7 |
|  | 3 | 21.5 | 15.9 |
|  | 4 | 15.9 | 12.4 |
|  | 5 | 7.1 | 17.6 |
|  | 6 | 8 | 12.2 |

| **Fig. 2** |  | Time (min) | | | | | | | | | | | |  | 0-10 min | 15-60 min | 15-30 min | 35-60 min |
| --- | --- | --- | --- | --- | --- | --- | --- | --- | --- | --- | --- | --- | --- | --- | --- | --- | --- | --- |
|  | mice | 5 | 10 | 15 | 20 | 25 | 30 | 35 | 40 | 45 | 50 | 55 | 60 |  | Phase1 | Phase 2 | Phase 2A | Phase 2B |
| Saline | 1 | 60 | 15 | 15 | 59 | 89 | 79 | 65 | 58 | 43 | 35 | 32 | 30 |  | 75 | 505 | 242 | 263 |
|  | 2 | 72 | 19 | 55 | 25 | 78 | 72 | 28 | 37 | 31 | 59 | 25 | 23 |  | 91 | 433 | 230 | 203 |
|  | 3 | 53 | 19 | 8 | 60 | 65 | 35 | 37 | 53 | 45 | 65 | 19 | 65 |  | 72 | 452 | 168 | 284 |
|  | 4 | 55 | 14 | 11 | 33 | 52 | 59 | 77 | 79 | 66 | 21 | 62 | 24 |  | 69 | 484 | 155 | 329 |
|  | 5 | 45 | 15 | 12 | 68 | 76 | 68 | 76 | 25 | 35 | 48 | 58 | 51 |  | 60 | 517 | 224 | 293 |
|  | 6 | 41 | 59 | 13 | 56 | 48 | 76 | 64 | 64 | 51 | 26 | 23 | 16 |  | 100 | 437 | 193 | 244 |
| NT | 1 | 36 | 5 | 6 | 25 | 35 | 31 | 64 | 73 | 76 | 72 | 65 | 29 |  | 41 | 476 | 97 | 379 |
|  | 2 | 35 | 16 | 22 | 36 | 67 | 72 | 69 | 30 | 56 | 63 | 19 | 23 |  | 51 | 457 | 197 | 260 |
|  | 3 | 61 | 4 | 4 | 18 | 27 | 29 | 46 | 72 | 8 | 37 | 55 | 62 |  | 65 | 358 | 78 | 280 |
|  | 4 | 64 | 3 | 2 | 22 | 58 | 15 | 8 | 71 | 59 | 41 | 35 | 16 |  | 67 | 327 | 97 | 230 |
|  | 5 | 43 | 12 | 7 | 35 | 28 | 38 | 19 | 38 | 55 | 69 | 59 | 13 |  | 55 | 361 | 108 | 253 |
|  | 6 | 38 | 6 | 8 | 39 | 22 | 75 | 77 | 24 | 55 | 12 | 18 | 24 |  | 44 | 354 | 144 | 210 |
| SR 48692+NT | 1 | 73 | 6 | 8 | 20 | 34 | 27 | 45 | 37 | 62 | 14 | 85 | 24 |  | 79 | 356 | 89 | 267 |
|  | 2 | 27 | 26 | 7 | 12 | 23 | 68 | 13 | 73 | 20 | 51 | 61 | 60 |  | 53 | 388 | 110 | 278 |
|  | 3 | 26 | 20 | 3 | 9 | 56 | 78 | 73 | 81 | 78 | 22 | 22 | 35 |  | 46 | 457 | 146 | 311 |
|  | 4 | 85 | 3 | 27 | 12 | 12 | 84 | 82 | 70 | 28 | 64 | 39 | 27 |  | 88 | 445 | 135 | 310 |
|  | 5 | 78 | 3 | 16 | 45 | 59 | 17 | 21 | 35 | 65 | 27 | 31 | 18 |  | 81 | 334 | 137 | 197 |
|  | 6 | 63 | 4 | 4 | 41 | 42 | 24 | 64 | 18 | 25 | 88 | 16 | 14 |  | 67 | 336 | 111 | 225 |
| SR 142948A+NT | 1 | 58 | 12 | 8 | 71 | 74 | 44 | 75 | 72 | 58 | 49 | 44 | 35 |  | 70 | 530 | 197 | 333 |
|  | 2 | 69 | 7 | 5 | 76 | 68 | 61 | 58 | 25 | 31 | 35 | 39 | 44 |  | 76 | 442 | 210 | 232 |
|  | 3 | 73 | 3 | 36 | 18 | 61 | 64 | 35 | 78 | 24 | 61 | 60 | 52 |  | 76 | 489 | 179 | 310 |
|  | 4 | 17 | 14 | 31 | 62 | 75 | 65 | 75 | 64 | 49 | 20 | 17 | 13 |  | 31 | 471 | 233 | 238 |
|  | 5 | 55 | 10 | 7 | 75 | 42 | 67 | 15 | 21 | 45 | 27 | 29 | 12 |  | 65 | 340 | 191 | 149 |
|  | 6 | 16 | 28 | 21 | 27 | 65 | 58 | 78 | 27 | 26 | 18 | 35 | 16 |  | 44 | 371 | 171 | 200 |

| **Fig.3A** |  | PWMT (g) | |
| --- | --- | --- | --- |
|  | mice | Prior | After |
| Sham+Saline | 1 | 0.6 | 1 |
|  | 2 | 0.6 | 1 |
|  | 3 | 1 | 0.6 |
|  | 4 | 1 | 1 |
|  | 5 | 1 | 1 |
|  | 6 | 1.4 | 1.4 |
| Sham+NT | 1 | 1 | 0.6 |
|  | 2 | 1 | 1.4 |
|  | 3 | 1 | 1.4 |
|  | 4 | 0.6 | 1 |
|  | 5 | 1 | 1.4 |
|  | 6 | 1 | 1 |
| CPNL+Saline | 1 | 0.16 | 0.04 |
|  | 2 | 0.02 | 0.04 |
|  | 3 | 0.008 | 0.02 |
|  | 4 | 0.16 | 0.04 |
|  | 5 | 0.07 | 0.16 |
|  | 6 | 0.02 | 0.008 |
| CPNL+NT | 1 | 0.04 | 0.6 |
|  | 2 | 0.16 | 1 |
|  | 3 | 0.04 | 0.4 |
|  | 4 | 0.04 | 1 |
|  | 5 | 0.16 | 0.6 |
|  | 6 | 0.008 | 0.4 |

| **Fig.3B** |  | PWMT (g) | |
| --- | --- | --- | --- |
|  | mice | Prior | After |
| Sham+SR48692+NT | 1 | 1 | 1 |
|  | 2 | 1.4 | 1 |
|  | 3 | 1 | 0.6 |
|  | 4 | 0.4 | 1 |
|  | 5 | 1 | 1 |
|  | 6 | 1 | 1 |
| Sham+SR142948A+NT | 1 | 1 | 1 |
|  | 2 | 1 | 0.6 |
|  | 3 | 1 | 1 |
|  | 4 | 1 | 1.4 |
|  | 5 | 0.6 | 1 |
|  | 6 | 0.6 | 0.6 |
| CPNL+SR48692+NT | 1 | 0.16 | 0.4 |
|  | 2 | 0.008 | 0.4 |
|  | 3 | 0.04 | 1 |
|  | 4 | 0.04 | 0.6 |
|  | 5 | 0.02 | 0.6 |
|  | 6 | 0.07 | 0.4 |
| CPNL+SR142948A+NT | 1 | 0.02 | 0.16 |
|  | 2 | 0.16 | 0.04 |
|  | 3 | 0.008 | 0.04 |
|  | 4 | 0.16 | 0.008 |
|  | 5 | 0.02 | 0.16 |
|  | 6 | 0.008 | 0.02 |

| **Fig.4A** |  | sEPSC frequency (Hz) | sEPSC amplitude (pA) |
| --- | --- | --- | --- |
| Baseline | 1 | 6.86 | 23.38 |
|  | 2 | 7.75 | 37.79 |
|  | 3 | 5.74 | 16.85 |
|  | 4 | 4.31 | 50.37 |
|  | 5 | 12.08 | 21.21 |
|  | 6 | 8.75 | 47.08 |
|  | 7 | 15.06 | 14.27 |
|  | 8 | 8.53 | 10.41 |
|  | 9 | 5.66 | 23.54 |
|  | 10 | 9.92 | 13.15 |
|  | 11 | 4.67 | 11.17 |
|  | 12 | 5.29 | 9.72 |
|  | 13 | 10.43 | 43.01 |
|  | 14 | 4.26 | 19.69 |
|  | 15 | 5.54 | 12.24 |
| NT 2μM | 1 | 7.42 | 20.95 |
|  | 2 | 6.15 | 48.86 |
|  | 3 | 5.24 | 20.99 |
|  | 4 | 10.53 | 39.48 |
|  | 5 | 6.06 | 8.55 |
|  | 6 | 12.39 | 15.12 |
|  | 7 | 3.16 | 47.34 |
|  | 8 | 4.12 | 11.23 |
|  | 9 | 4.33 | 43.06 |
|  | 10 | 5.78 | 31.05 |
|  | 11 | 7.86 | 19.46 |
|  | 12 | 14.92 | 21.77 |
|  | 13 | 10.88 | 17.11 |
|  | 14 | 9.37 | 20.57 |
|  | 15 | 5.34 | 13.15 |

| **Fig.4B** |  | sIPSC frequency (Hz) | sIPSC amplitude (pA) |
| --- | --- | --- | --- |
| Baseline | 1 | 2.23 | 77.93 |
|  | 2 | 1.42 | 25.14 |
|  | 3 | 2.35 | 23.97 |
|  | 4 | 1.09 | 89.36 |
|  | 5 | 3.64 | 35.78 |
|  | 6 | 1.71 | 24.4 |
|  | 7 | 2.69 | 70.64 |
|  | 8 | 5.28 | 38.99 |
|  | 9 | 1.88 | 21.84 |
|  | 10 | 1.15 | 20.41 |
| NT 2μM | 1 | 4.29 | 29.94 |
|  | 2 | 3.36 | 82.81 |
|  | 3 | 7.06 | 44.47 |
|  | 4 | 1.34 | 75.14 |
|  | 5 | 5.69 | 20.23 |
|  | 6 | 8.52 | 26.01 |
|  | 7 | 2.83 | 78.36 |
|  | 8 | 3.96 | 26.22 |
|  | 9 | 7.67 | 31.82 |
|  | 10 | 2.08 | 25.62 |

| **Fig.4C** | | mIPSC frequency (Hz) | mIPSC amplitude (pA) |
| --- | --- | --- | --- |
| Baseline | 1 | 1.18 | 45.62 |
|  | 2 | 2.31 | 24.28 |
|  | 3 | 1.46 | 56.65 |
|  | 4 | 1.59 | 32.33 |
|  | 5 | 2.24 | 37.92 |
|  | 6 | 2.55 | 25.59 |
|  | 7 | 1.91 | 48.81 |
|  | 8 | 2.64 | 39.32 |
|  | 9 | 1.31 | 28.45 |
|  | 10 | 1.88 | 41.07 |
|  | 11 | 1.15 | 21.5 |
| NT 2μM | 1 | 3.44 | 36.23 |
|  | 2 | 2.47 | 29.88 |
|  | 3 | 5.12 | 41.42 |
|  | 4 | 6.63 | 35.79 |
|  | 5 | 4.28 | 24.48 |
|  | 6 | 1.95 | 34.07 |
|  | 7 | 4.85 | 26.43 |
|  | 8 | 3.96 | 22.95 |
|  | 9 | 2.71 | 61.52 |
|  | 10 | 5.8 | 36.57 |
|  | 11 | 4.35 | 54.46 |

| **Fig.4D** | | mIPSC frequency (Hz) | mIPSC amplitude (pA) |
| --- | --- | --- | --- |
| Baseline | 1 | 3.19 | 82.55 |
|  | 2 | 1.58 | 45.98 |
|  | 3 | 2.51 | 76.09 |
|  | 4 | 2.6 | 60.54 |
|  | 5 | 3.39 | 105.93 |
|  | 6 | 4.34 | 51.17 |
|  | 7 | 3.95 | 58.69 |
|  | 8 | 3.26 | 66.23 |
|  | 9 | 2.48 | 75.22 |
| NT 2μM | 1 | 4.29 | 69.48 |
|  | 2 | 3.36 | 100.16 |
|  | 3 | 7.06 | 58.7 |
|  | 4 | 1.34 | 44.03 |
|  | 5 | 5.69 | 81.95 |
|  | 6 | 6.52 | 59.49 |
|  | 7 | 3.83 | 62.63 |
|  | 8 | 2.67 | 98.32 |
|  | 9 | 6.08 | 59.44 |

| **Fig.5A** |  | GABAergic frequency (Hz) | Glyergic frequency (Hz) |
| --- | --- | --- | --- |
| Baseline | 1 | 2.53 | 2.95 |
|  | 2 | 1.24 | 8.47 |
|  | 3 | 2.93 | 3.33 |
|  | 4 | 4.82 | 1.81 |
|  | 5 | 5.25 | 12.24 |
|  | 6 | 1.18 | 8.29 |
|  | 7 | 1.6 | 7.91 |
|  | 8 | 2.91 | 6.25 |
|  | 9 | 1.54 | 2.66 |
|  | 10 | 7.29 | 1.74 |
|  | 11 | 2.41 | 9.06 |
|  | 12 | 1.3 | 1.55 |
| NT 2μM | 1 | 1.66 | 7.72 |
|  | 2 | 4.18 | 9.26 |
|  | 3 | 1.09 | 14.2 |
|  | 4 | 2.54 | 1.28 |
|  | 5 | 2.84 | 12.97 |
|  | 6 | 7.57 | 1.94 |
|  | 7 | 1.56 | 2.23 |
|  | 8 | 1.32 | 3.47 |
|  | 9 | 1.29 | 1.2 |
|  | 10 | 6.22 | 1.91 |
|  | 11 | 1.38 | 9.88 |
|  | 12 | 1.04 | 8.29 |

| **Fig.5B** |  | GABAergic frequency (Hz) | Glyergic frequency (Hz) |
| --- | --- | --- | --- |
| Baseline | 1 | 1.57 | 4.32 |
|  | 2 | 1.32 | 2.02 |
|  | 3 | 2.33 | 9.38 |
|  | 4 | 1.01 | 9.1 |
|  | 5 | 1.89 | 6.58 |
|  | 6 | 5.04 | 7.42 |
|  | 7 | 1.44 | 2.47 |
|  | 8 | 2.54 | 8.85 |
|  | 9 | 1.06 | 6.79 |
|  | 10 | 4.67 | 11.65 |
|  | 11 | 1.14 | 4.16 |
|  | 12 | 9.85 | 2.22 |
|  | 13 | 2.75 | 2.61 |
|  | 14 | 1.97 | 5.42 |
| NT 2μM | 1 | 8.24 | 3.06 |
|  | 2 | 11.75 | 7.32 |
|  | 3 | 4.54 | 10.41 |
|  | 4 | 1.49 | 10.12 |
|  | 5 | 4.12 | 6.52 |
|  | 6 | 2.66 | 9.65 |
|  | 7 | 4.67 | 6.52 |
|  | 8 | 1.96 | 9.32 |
|  | 9 | 9.35 | 3.44 |
|  | 10 | 3.79 | 9.76 |
|  | 11 | 2.18 | 13.36 |
|  | 12 | 9.22 | 3.32 |
|  | 13 | 7.43 | 9.23 |
|  | 14 | 6.52 | 8.87 |
