# Supplementary figures and images for: Neurotensin Attenuates Nociception by Facilitating Inhibitory Synaptic Transmission in the Mouse Spinal Cord
Source: Front Neural Circuits. 2021 Dec 24;15:775215. doi: 10.3389/fncir.2021.775215 (PMC8740200; doi:10.3389/fncir.2021.775215)

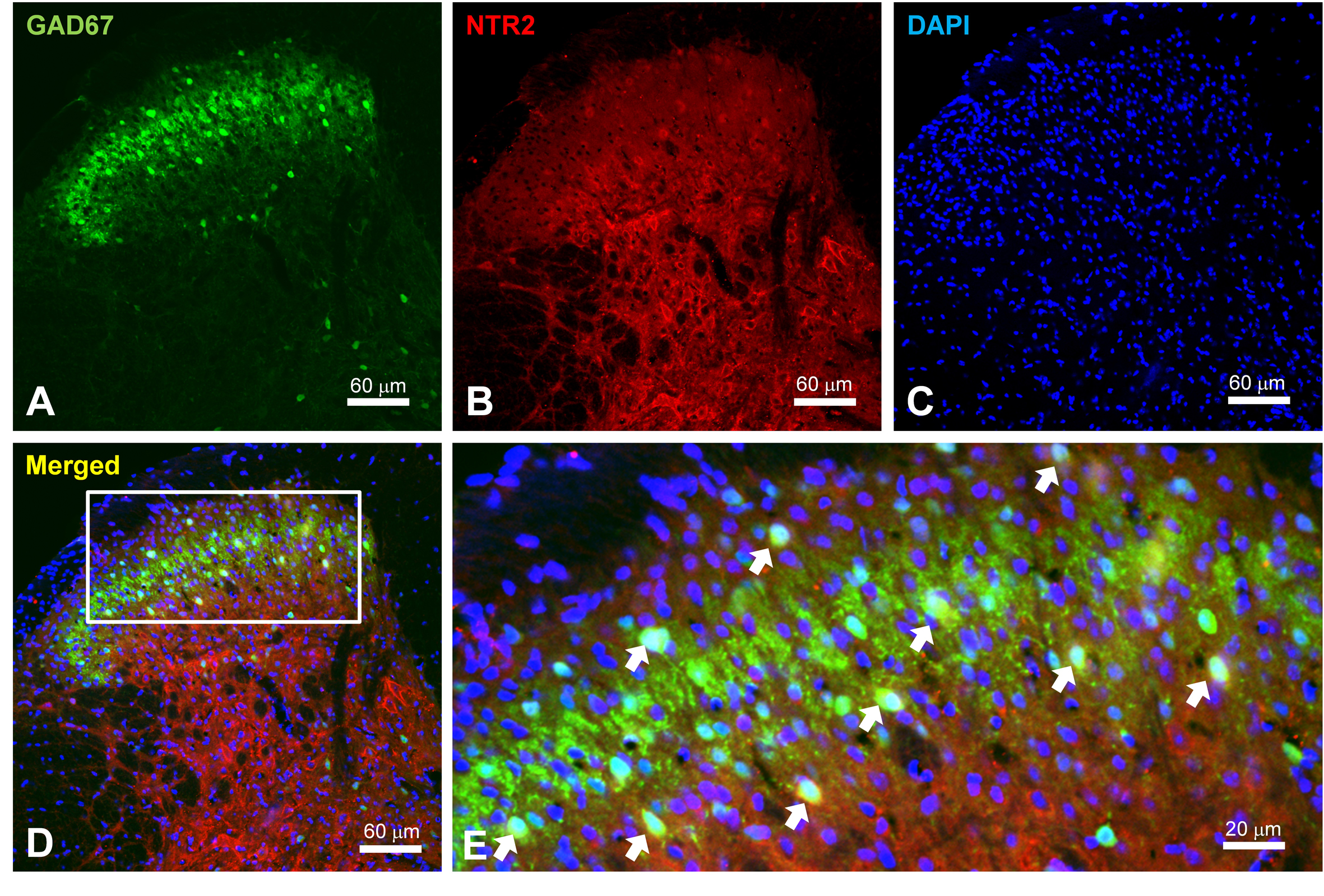

Supplement: Supplementary file 2 [file Image_1.jpg]
